# Global Health Education: a cross-sectional study among German medical students to identify needs, deficits and potential benefits (Part 1 of 2: Mobility patterns & educational needs and demands)

**DOI:** 10.1186/1472-6920-10-66

**Published:** 2010-10-08

**Authors:** Kayvan Bozorgmehr, Kirsten Schubert, Johannes Menzel-Severing, Peter Tinnemann

**Affiliations:** 1 Institute for Social Medicine, Epidemiology and Health Economics, Charité - University Medical Center, Berlin, Germany; 2 Globalisation and Health Initiative (GandHI), German Medical Students' Association (bvmd), Bonn, Germany

## Abstract

**Background:**

In recent years, education and training in global health has been the subject of recurring debate in many countries. However, in Germany, there has been no analysis of the educational needs or demands of medical students, or the educational deficits or potential benefits involved in global health education. Our purpose is to analyse international health elective patterns of medical students enrolled at German universities and assess whether or how they prepare for their electives abroad. We examine the exposure of medical students enrolled at German universities to training courses in tropical medicine or global health and assess students' perceived needs and demands for education in global health.

**Methods:**

Cross-sectional study among medical students in Germany including all 36 medical schools during the second half of the year 2007. All registered medical students were eligible to participate in the study. Recruitment occurred via electronic mailing-lists of students' unions. We developed a web-based, semi-structured questionnaire to capture students' international mobility patterns, preparation before electives, destination countries, exposure to and demand for global health learning opportunities.

**Results:**

1126 online-replies were received and analysed from all registered medical students in Germany (N = 78.067). 33.0% of all respondents (370/1126) declared at least one international health elective and of these, 36.0% (133/370) completed their electives in developing countries. 36.0% (131/363) did not prepare specifically at all, 59.0% (214/363) prepared either by self-study or declared a participation in specific preparation programmes. 87.8% of 5^th ^and 6^th ^year students had never participated in a global health course and 72.6% (209/288) had not completed a course in tropical medicine. 94.0% (861/916) endorsed the idea of introducing global health into medical education.

**Conclusion:**

Students in our sample are highly mobile during their studies. International health electives are common, also in developing countries. Formal preparation beyond self-study is virtually non-existent amongst our sample and the participation rate in courses of tropical medicine or global health is appallingly low. We have identified unmet perceived needs and the demand for more learning opportunities in global health in our sample, urging for reforms to adjust curricula to a globalising world.

## Background

Over the past three decades, globalisation has reduced barriers to transnational contact and enabled people to engage with each other physically, legally, culturally, and psychologically in one world [1]. It is widely acknowledged that globalisation affects the health of populations and individuals through a variety of determinants including changes in market structures, communication, diffusion of information, cross-cultural interaction, environmental change and mobility [2].

At the same time, global health has gained relevance as a 'field of practice, research and education focussed on health and the social, economic, political and cultural forces that shape it across the world' *(Rowson M, Hughes R, Smith A, Maini A, Martin S, Miranda JJ, Pollit V, Wake R, Willott C, Yudkin JS: Global Health and medical education - definitions, rationale and practice, unpublished)*. Although the discipline has an historical association with the distinct needs of developing countries, it is especially concerned with health-related issues that transcend national boundaries and with the impacts of globalisation *(ibid., unpublished)*. This makes it distinctly different to the discipline 'international health', which is often inaccurately used interchangeably.

The implications of perceiving health as a global issue have been a subject of debate, particularly in medical education [3-6]. Through the years, calls for more training and opportunities in global health for the health workforce have become louder [7]; with supporters bringing in many plausible reasons why global health should be included or its place strengthened in medical education.

There is evidence to suggest a high international mobility of medical students during their training and an increasing proportion completing their electives abroad. Figures recently published in the United States of America (US) show that the percentage of US medical students participating in international rotations has increased steadily over the last 30 years [8], while in the United Kingdom (UK) 40 per cent of medical students visit a developing country in their elective period [9]. Miranda et al. note a lack of preparation and education for medical students in the UK before their elective abroad, apart from individual advice about occupational and travel health risk assessments [9]. Therefore recommendations for the delivery of preparational modules embedded in a comprehensive programme of teaching international health have been formulated [10].

Apart from pre-elective training, studies to analyse the prevalence of learning opportunities in international or global health at medical schools have been conducted nationally in recent years, e.g. in the UK *(Medsin: Medsin Global Health Survey, unpublished)*, Canada [11] or in Italy [12], as well as internationally *(Rowson M, Hughes R, Smith A, Maini A, Martin S, Miranda JJ, Pollit V, Wake R, Willott C, Yudkin JS: Global Health and medical education - definitions, rationale and practice, unpublished. Sundell T, Ashorn P: International Health in Medical Curricula: Report of an International Survey, unpublished)*. These studies are mostly available as grey literature. In a few studies, medical students and their interest in international or global health have been the subject of interest. In 2003 Matin conducted a study among 45% (n = 1284) of all medical students actively studying at Finnish medical schools and found a high interest in global health education, with 77.4% of the respondents stating that global health subjects should be included in the compulsory curriculum of all medical students (*unpublished*). Another study conducted among 310 of more than 2500 students at the King's College in London (UK) identified a high level of dissatisfaction with the provision of global health teaching (76% were dissatisfied) and a high enthusiasm (92%) to study it in the future [13].

Data on German medical students' mobility is patchy, while data on the prevalence of global health education or students' interest in global health is not available.

Regarding medical students' mobility, available data and research done on students' educational needs in international or global issues is rare *(Bozorgmehr K, Tinnemann P: The State of Global Health in German Medical Education: a systematic review, unpublished)*. An alumni survey in Germany has shown that 60% of alumni (out of 4720) have been abroad at least once during their medical studies [14], not specifying preparation, destination or purpose. Kuhlmey and Dettmer found that among a sample of 357 1^st ^year medical students nearly 63.0% opt to work abroad, mainly in countries with better working conditions [15]. Representative data collections of the German Academic Exchange Service (*Deutscher Akademischer Austausch Dienst; DAAD*) reveal that 25% of all German medical students partially study abroad during their studies for a certain period [16], again without specifying preparation or destinations. The mobility during medical studies is - at least partially - politically mediated and supported. For example, the student exchange programs of the German Medical Students' Association (*Bundesvertretung der Medizinstudierenden e.V*.) is financially supported with an annual budget of more than 200.000 Euros *(bvmd: Annual budget of the Exchange-Section of the German Medical Students' Association, personal communication) *provided for travel grants only by the Federal Foreign Office (*Auswärtiges Amt*).

However, the educational needs of German medical students in the context of globalisation have not been analysed yet *(Bozorgmehr K, Tinnemann P: The State of Global Health in German Medical Education: a systematic review, unpublished)*. It is not yet known how many medical students enrolled at German universities complete their electives abroad and in which countries they do their international health elective (IHE). Moreover, nothing is known about students' pre-elective preparations and no studies exist which analyse their exposure to courses of tropical medicine or global health.

Moreover, two international surveys which have analysed the prevalence of international health *(Sundell T, Ashorn P: International Health in Medical Curricula: Report of an International Survey, unpublished) *or global health *(Rowson M, Hughes R, Smith A, Maini A, Martin S, Miranda JJ, Pollit V, Wake R, Willott C, Yudkin JS: Global Health and medical education - definitions, rationale and practice, unpublished) *at medical schools have produced unsatisfactory results in the case of Germany, with no universities responding to one *(Rowson M, Hughes R, Smith A, Maini A, Martin S, Miranda JJ, Pollit V, Wake R, Willott C, Yudkin JS: Global Health and medical education - definitions, rationale and practice, unpublished)*, and only 6 responding to the other *(Sundell T, Ashorn P: International Health in Medical Curricula: Report of an International Survey, unpublished)*. The last study which analysed the 'extent to which issues of tropical medicine and public health in developing countries' are represented in German medical education was conducted in 1995 *(Stich A, Köbler C, Strauß R, Hampel D, Fleischer K: Tropenmedizinische Ausbildung in Deutschland - Erfolge und Defizite: Teil 1-Lehrveranstaltungen zum Themenbereich "Tropenmedizin und Gesundheitsversorgung in Entwicklungsländern" an deutschen medizinischen Fakultäten, unpublished)*. Since then, there have been no further studies analysing the prevalence of global health learning opportunities at German medical schools *(Bozorgmehr K, Tinnemann P: The State of Global Health in German Medical Education: a systematic review, unpublished) *or the demand therefore such opportunities among medical students enrolled in Germany.

### Purpose of this study

1. To analyse the international health elective (IHE) patterns of medical students enrolled at German universities and to assess whether or how they prepare for their IHE.

2. To examine the exposure of medical students enrolled at German universities to courses in tropical medicine and global health.

3. To assess the prevalence of global health education at respondents' medical schools as well as perceived needs and demands for education in global health.

## Methods

### Study Design

Nationwide, cross-sectional study conducted between May and December 2007 by the Globalisation and Health Initiative (GandHI) of the German Medical Students' Association (*Bundesvertretung der Medizinstudierenden e.V*.) (bvmd).

We created a web-based, semi-structured questionnaire with 28 questions (25 structured, 3 open) in German language, of which 18 addressed the purposes of this paper. These questions captured student demographics, experiences abroad, destination countries, type of preparation before the IHE and exposure to relevant educational interventions (i.e. participation in courses of global health or tropical medicine) (see Questionnaire Outline). The survey was anonymous and participants gave their informed consent for participation. The answers and the identity of the respondents cannot be connected. Ethical approval for this study was exempt according to section (§) 15 of the professional code of conduct of the Medical Council of Berlin.

### Recruitment

Using electronic mailing-lists of German students' unions, medical students from all 36 German medical schools were invited by e-mail to complete the online-survey. In addition, internet links were established on the website of the German Medical Students' Association http://www.bvmd.de and on the website of a German medical students' journal http://www.aerzteblatt-studieren.de/. All registered medical students were eligible to participate in the study, but registration validity was not checked before filling in the online questionnaire.

The call for participation simply contained a contextual reference to issues of medical education. To reduce selection-bias we avoided any words in the announcement which could be associated with global health, globalisation, development aid, development cooperation, international health or public health.

### Questionnaire Outline

The questionnaire consisted of five different blocks of questions, of which three address the issues presented in this paper.

#### 1. Student mobility

Student mobility was captured by a filter question (yes/no) on students' participation in IHE up to now.

##### Destinations of IHE

Respondents who stated having completed an IHE were asked three further questions to specify the destination country and the purpose of their first, second and/or third IHE. Three free text answer options were provided to specify the destination countries.

##### Purposes of IHE

Multi-option answers were provided to specify the respective purposes, consisting of 'elective rotation' *(Famulatur) *and 'senior clerkships' *(Praktisches Jahr - rotations to complete in the final clinical year)*, with a free text option for 'other purposes'.

##### Preparation before IHE

To capture the actual type of medical students' preparation before their IHE, a set of multi-option answers was provided including "literature studies (except travel guides)", "no specific preparation" and a field for free text answers to specify potential preparation courses or other types of preparation. These were summarized under the category "courses" and "self-study". The response option "no specific preparation" was treated as an exclusive category. Respondents who provided contradictory statements were excluded from further analysis in this section.

A maximum of three study-related transborder movements per participant were thus captured. Respondents who declared no IHE were asked in a closed-ended question ('yes/no/not sure') whether they are currently planning a stay abroad.

#### 2. Exposure to courses in tropical medicine or global health

The questionnaire captured other relevant educational interventions beyond immediate preparation before IHE, such as participation in courses of global health or tropical medicine. The general exposure to these interventions was captured by two yes/no questions, namely 'Have you ever participated in a global health course?' and 'Have you ever participated in a tropical medicine course?'.

#### 3. Prevalence of education in global health and perceived needs and demands

To capture the prevalence of education in global health, we provided a closed-ended question ('Is there any education in such global health issues at your medical school?') with the response options 'yes/no/no idea (*weiß nicht*)'. Those who answered this question with 'yes', where further asked to specify the form of courses.

Perceived needs regarding the prevalence of global health courses at students' institution was captured by the closed-ended question 'In your opinion, is the existing supply [of global health course opportunities] sufficient?'; with the response options 'yes/no/no preference *(kann ich nicht beurteilen)*'.

Participants were then further asked: 'Would you endorse the introduction of global health learning opportunities?'; along with a set of response options being 'yes, as compulsory courses/yes, as elective compulsory courses/yes, as optional courses/no/no preference (*dazu habe ich keine Meinung)*'.

According to the German Licensing Regulations for physicians [17], 'compulsory courses' (*Pflichtfächer*) are courses which must be completed by all medical students. 'Elective compulsory courses' *(Wahlpflichtfächer) *are characterised by being compulsory and covering a wide range of topics among which students can choose at least one according to their interest. The completion of an 'optional course' (*freiwilliges Zusatzangebot*) in contrast is completely voluntary and as such optional.

In order to reduce misunderstandings caused by the translation process, the original questions in German language related to this section are included in brackets within the figures of the result section.

The term 'tropical medicine course' was not explicitly defined, since tropical medicine is an established term and a distinct subject known to medical students.

To clarify the difference between tropical medicine and the topics deemed to fall under the subject heading 'global health', we used an operational definition of 'global health course' (*in German: "Globale Gesundheit"*). In the questionnaire, we defined 'global health courses' as 'courses in which students analyse the influence on people's health of factors such as poverty, debt, globalisation, health systems and health financing, human rights, hunger, armed conflicts and migration'.

#### Demographics

A third block captured student demographics, including age, level of study and university affiliation.

### Stratification

For the purposes of this study, respondents were stratified into subgroups according to the criteria shown as variables "Student Mobility" and "Educational Intervention" in Table 1. In order to group the respondents depending on the destination country of their IHE, a new variable was assigned to each declared destination country categorising it as either an industrialised or developing country. Depending on the frequency of completed IHE, we received different mobility patterns and categorised students into groups who completed their IHE predominantly in developing countries (IHE-South) or predominantly in industrialised countries (IHE-North) (see Table 1). A developing country was defined as a country with a Human Development Index less than 0.85.

**Table 1:** Stratification Criteria

Stratification criteria	
**Variable**	**Criteria**

**Student mobility**	

IHE-Yes	Any respondent declaring **at least one **study-related trans-border movement

IHE-No	Any respondent declaring **no **study-related trans-border movement

IHE-North	Destination countries of international health electives (IHE) are **pre-dominantly industrialised **countries. (Eligible combinations: n, nn, snn, nns, nsn, nnn)*

IHE-South	Destination countries of international health electives (IHE) are **pre-dominantly developing **countries. (Eligible combinations: s, ss, sn, ns, ssn, sns, nss, sss)*

**Exposure to educational interventions**	

GH-yes	Any respondent declaring a **participation **in global health courses.

GH-no	Any respondent declaring **no participation **in global health courses.

TM-yes	Any respondent declaring a **participation **in courses of tropical medicine.

TM-no	Any respondent declaring **no participation **in courses of tropical medicine.

* A variable was added to each destination country; 'n' if the destination country was industrialised, 's' if the destination country was a developing country. A developing country was defined as a country with a Human Development Index < 0.85

### Statistical Analysis

The distribution of categorical and ordinal data is described with absolute and relative frequency. The differences in distributions of findings between subgroups were analysed with Fischer's exact test for categorical data due to the small sample sizes of subgroups. All tests were performed two-tailed; the level of significance was set at α = 0.05. Analyses were done with SAS version 9.1., graphs were created with Microsoft Excel and Adobe Illustrator.

## Results

Students from all 36 medical schools replied, resulting in N = 1126 filled-out online questionnaires. This constitutes an overall response rate of 1.4% from all medical students enrolled during the winter term 2007/2008 in Germany (N = 78.067) [18]. 77.6% of all responses (874/1126) were from eight universities, the other 22.4% of responses were received from the remaining 28 universities. (Additional File 1: Annex 1-University affiliation)

The proportion of responding students from pre-clinical terms (1^st ^and 2^nd ^year), early clinical terms (3^rd ^and 4^th ^year) and final clinical terms (5^th ^and 6^th ^year and above) was nearly equal (Table 2).

**Table 2:** Baseline characteristics and stratification of the study population

Baseline characteristics and stratification of the study population (N = 1126)	
**Variable**	

**Demographics**	

Age (yrs) (M ± SD)	23.5 ± 2.4 (n = 1096)

Age > 30 yrs	3.0% (n = 30)

	

Level of study (terms) (M(SD)	7.0 ± 3.4 (N = 1126)

Term 1-4 (1st and 2nd year)	31.2% (351/1126)

Term 5-8 (3rd and 4th year)	37.7% (425/1126)

Term 9-12 (5th and 6th year)	25.6% (288/1126)

Terms above 12	5.5% (62/1126)

	

**Student Mobility**	

International health electives (IHE)	

IHE-yes *(all terms)*	33.0% (370/1126)

*Frequency of participation in IHE*	

Once	60.0% (221/370)

Twice	25.0% (92/370)

Three times	15.0% (57/370)

	

IHE-no *(all terms)*	67.0% (756/1126)

	

**Exposure to educational interventions**	

*Participation in Global Health (GH) courses*	

GH-yes *(all terms)*	9.0% (106/1126)

GH-no *(all terms)*	91.0% (1020/1126)

	

*Participation in Tropical Medicine (TM) courses*	

TM-yes *(all terms)*	16.0% (175/1126)

TM-no *(all terms)*	84.0% (951/1126)

### 1. Student Mobility

**Figure 1:**
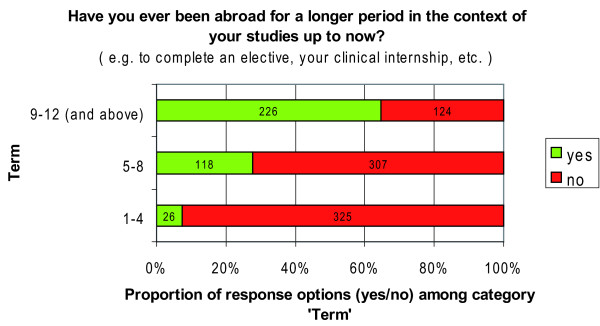
**Students' mobility by level of study**.

To avoid an artificially low mobility rate due to students of all years reporting on their international electives, we stratified students' mobility by level of study. This procedure produced a 65.0% mobility rate for 5^th ^and 6^th ^year students (terms 9-12 and above) (Figure 1).

**Figure 2:**
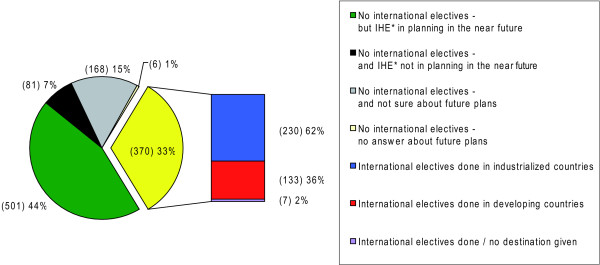
**Past and future mobility**. *IHE = international health elective.

Of all respondents, one third (370/1126) had already gained experiences abroad at least once, while two thirds (756/1126) stated no participation in IHE. Of those with experiences abroad, 60.0% (221/370) had been abroad once, 25.0% (92/370) twice and 15.0% (57/370) three times, indicating a high mobility (Table 2). 66.0% (501/756) of those respondents without experiences abroad had concrete plans for an IHE in the near future (Figure 2), indicating a high potential for future mobility.

#### Destinations of IHE

12.0% (133/1126) of all respondents, i.e. 36.0% (133/370) of those students with experiences abroad completed their IHE predominantly in developing countries. The proportion of these students ranged from 35.0% among 5^th ^and 6^th ^year students to 42.0% among 1^st ^and 2^nd ^year students.

20.0% (230/1126) of all respondents, i.e. 62.0% (230/370) of those with experiences abroad, mainly completed their IHE in industrialised countries (Figure 2). Seven respondents gave no information about their destination countries and were excluded for further stratification in statistical tests.

**Figure 3:**
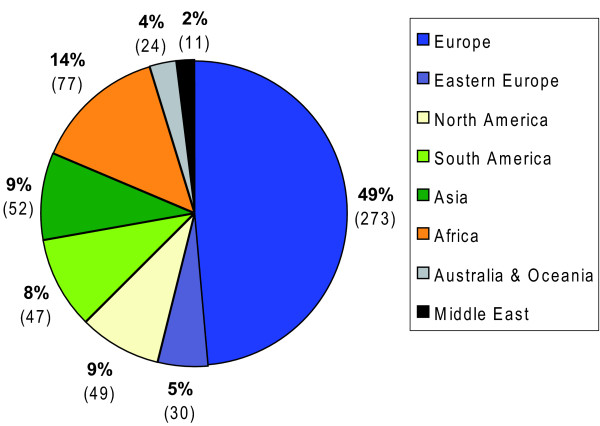
**Destination of international health electives by continent and region**.

When analysing the overall destinations of IHE by continent and region, we found that the majority of IHE (63.0%) took place among Europe, Eastern Europe and North America, while one third (31.0%) completed their IHE in African, Asian and South American countries (Figure 3).

The top five destinations among Europe and North America were Switzerland (n = 57; 10.1%), the USA (n = 42; 7.4%), Spain (n = 40; 7.1%), Austria (n = 39; 6.9%) and France (n = 26; 4.6%). As for the African, Asian and South American continents, the top five destinations were India (n = 18; 3.2%), South Africa (n = 17; 3.0%), Tanzania (n = 16; 2.8%), Mexico (n = 14; 2.5%) and Nepal (n = 8; 1.4%).

#### Purposes of IHE

The German Licensing Regulations for physicians specify several practical stages during medical studies, which can be completed partially or totally abroad: a three month mandatory nursing period *(Krankenpflegepraktikum) *as part of preclinical studies; four one-month clinical electives *(Famulatur) *during clinical studies; and a one-year clinical internship or 'senior clerkship' in the final clinical year *(Praktisches Jahr) *[19].

**Figure 4:**
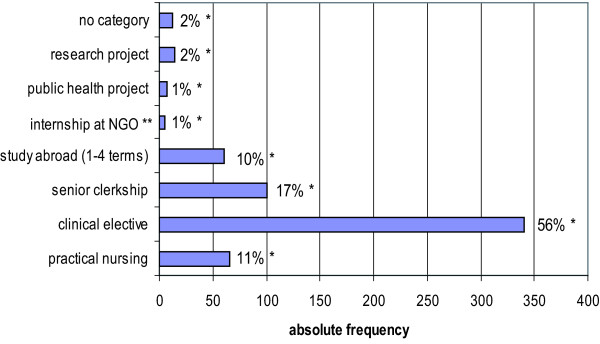
**Purposes of study-related transborder movements**. * Percentages refer to the proportion of categories among n = 604 answers. ** NGO = non-governmental organization.

Given the possibility of multi-option and open answers, 604 statements were made by 363 respondents to specify their purposes for study-related transborder movements. We summarised these according to the categories listed in Figure 4. International health electives, including compulsory 'practical nursing periods' in pre-clinical terms (66; 11.0%), 'clinical electives' in clinical study periods (340; 56.0%) and 'senior clerkships' or internships in final clinical terms (100; 17.0%) made up 84.0% (506/604) of the reasons for a study-related transborder movement in our sample. (Figure 4)

#### Preparation before IHE

Students in our sample had contacts with various health care systems (Figure 3). In response to the question of how they prepared themselves before their IHE, 36.0% (131/363) answered that they did not prepare specifically at all. Among these students 81.0% (106/131) had completed their IHE predominantly in industrialised countries. Eighteen respondents were excluded due to contradictory statements provided. Of those 214 students who declared preparations, the majority of 79.0% prepared by literature studies, followed by other types of self-study. 23.0% (50/214) had participated in preparation courses, either provided by universities or student-led (Table 3).

**Table 3:** Type of preparation before IHE

Type of preparation					
**Exclusive categories**					

	**N = 363 students (100%)**	**IHE-North (n = 230)**	**IHE-South (n = 133)**	**Line Total**	%* of N

	No specific preparation	106	25	**131**	**36%**

	Contradictory statements	11	7	**18**	**5%**

	Total N of replies in exclusive categories (column total)	117	32	**149**	**41%**

					

**Multi-option categories**					

	**N = 214 students (100%)**	**IHE-North (n = 113)**	**IHE-South (n = 101)**	**Line Total**	%* of N

**Self study**	Literature studies (except travel guides)	88	82	**170**	**79%**
	
	Personal communications/Interpersonal exchange of experience	10	14	**24**	**11%**
	
	Internet	8	13	**21**	**10%**
	
	Reports (elective reports etc.)	8	2	**10**	**5%**

					

**Courses**	Student organised	3	5	**8**	**4%**
	
	University provided	1	8	**9**	**4%**
	
	Language	21	7	**28**	**13%**
	
	Other courses	-	5	**5**	**2%**

	Other types of preparation/not categorisable	10	3	**13**	**6%**

	Total N of replies in multi-option categories (column total)**	149	138	**288**	**135%**

* Percentages refer to N = 363 and N = 214 students in exclusive and mutli-option categories respectively

**column total > 100% due to multi-option answers

### 2. Exposure to courses in tropical medicine or global health

Of all responding students 91.0% (1020/1126) had never participated in a global health course and 84.0% (951/1126) had never completed a course in tropical medicine (Table 2). A significantly higher proportion of 3^rd ^and 4^th ^year students (terms 5-8) and 5^th ^and 6^th ^year students (terms 9-12 and above) participated in global health (p = 0.002) or tropical medicine courses (p < 0.0005), while 1^st ^and 2^nd ^year students (terms 1-4) were represented significantly less among the course participants (Table 4).

**Table 4:** Course participation by level of study

Participation in courses of global helath and tropical medicine by level of study						
		**Course participation**				
		
		**yes**		**no**		

**Course**	**term**	**Abolute freq.**	%*	**Absolute freq.**	%*	p-value**

**Global Health**	1-4	17	4.8	334	95.2	**0.002**
		
	5-8	45	10.6	380	89.4	
		
	9-12	35	12.2	253	87.8	
	
	> 12	9	14.5	53	85.4	

**Tropical Medicine**	1-4	8	2.3	343	97.7	**< 0.0005**
		
	5-8	68	16.0	357	84.0	
		
	9-12	79	27.4	209	72.6	
	
	> 12	20	32.3	42	67.7	

*Percentages refer to the proportion of „Course participation yes/no" among the subgroup "term" (line total = 100%).

**p-value of Fisher's exact test was calculated for terms 1-4, 5-8 and 9-12.

Among the 5^th ^and 6^th ^year students, i.e. among nearly graduating students, 87.8% (253/288) had never participated in a global health course and 72.6% (209/288) had not even once participated in a course of tropical medicine (Table 4).

78.6% (885/1126) of all participants had neither participated in a global health course, nor completed a course in tropical medicine (Table 5). For participants in global health and tropical medicine, we found a slight overlap between the cohorts. 22.9% (40/175) of all students who participated in courses of tropical medicine also participated in a global health course (Table 5). Nevertheless, the cohorts were not identical, which is important to note for the analyses done in the second part of this series [20].

**Table 5:** Overlap of participants in courses of global health and tropical medicine

Frequency of participation in global health courses by participation in courses of tropical medicine					
		**Global Health course**			
		
		**yes**		**no**	
		
		**Absolute freq.**	%*	**Absolute freq.**	%*

**Tropical medicine course**	**yes**	40	22.9	135	77.1
	
	**no**	66	6.9	885	93.1

*Percentages refer to the proportion of "Global Health course yes/no" among the subgroup „Tropical medicine course yes/no" (line total = 100%).

**Figure 5:**
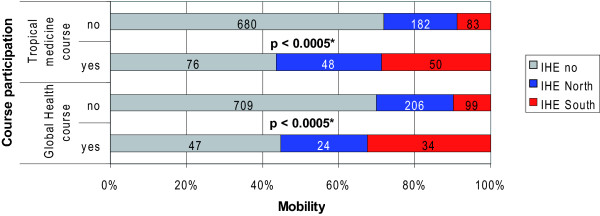
**Course participation and mobility**. *p-value of Fischer's exact test Percentages on the y-axis refer to the proportion of "IHE no", "IHE-North" and "IHE-South" among the subgroups „Global Health course yes/no" and "Tropical medicine course yes/no" (column total: 100%). Figures within each bar are absolute frequencies. Missing figures to N = 1126 are due to the exclusion of n = 7 respondents, who did not specify their IHE destinations. IHE = international health elective; IHE-South = electives predominantly completed in developing countries; IHE-North = electives predominantly completed in industrialised countries; IHE-no = no electives abroad.

The participation rate in courses of global health or tropical medicine was significantly (p < 0.0005) higher among students who completed their IHE predominantly in developing (IHE-South) than in industrialised countries (IHE-North) (Figure 5). The participation rate in these courses among the latter subgroup (IHE-North) was significantly (p < 0.0005) higher than among respondents who declared no IHE at all (IHE-no).

### 3. Prevalence of and demand for education in global health

16.0% (184/1126) of all respondents answered the question 'Is there any education in such global health issues at your medical school?' with 'yes', 36.0% (408/1126) declared 'no' and the majority of 47.0% (534/1126) had 'no idea' about the prevalence.

**Figure 6:**
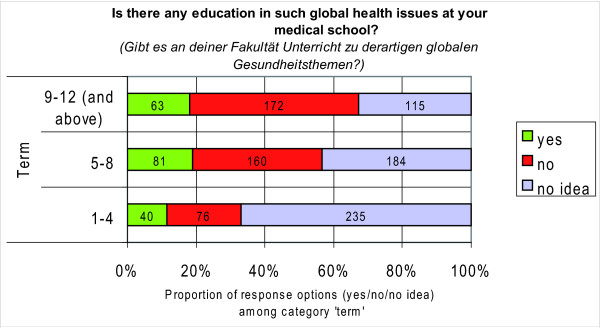
**Prevalence of global health education by level of study**.

The proportion of those who responded with 'yes' ranged between 11.0% and 19.0% among students from first to final years (Figure 6). The proportion of those who responded with 'no' increased considerably from 22.0% among 1^st ^and 2^nd ^year students (terms 1-4) to 49.0% among 5^th ^and 6^th ^year students (terms 9-12 and above). The proportion of those who had 'no idea' about their curricular offerings nearly halved and fell from 67.0% among freshman students to 33.0% among graduating students (Figure 6).

**Figure 7:**
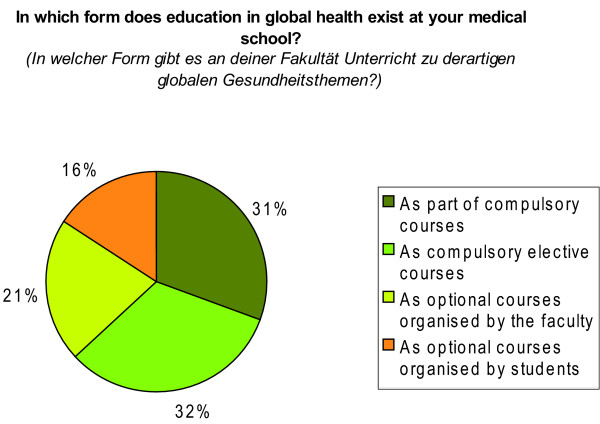
**Form of education in global health**. Percentages refer to N = 251 answers of 184 students (Total > 100% due to the possibility of multi-option answers).

The 184 students who answered the questions with 'yes' were from 25 different universities. At these universities, it was claimed by respondents that education in global heath existed mainly 'as compulsory elective course' or 'as part of compulsory courses', followed by optional courses organised by the faculty or by students. (Figure 7)

**Figure 8:**
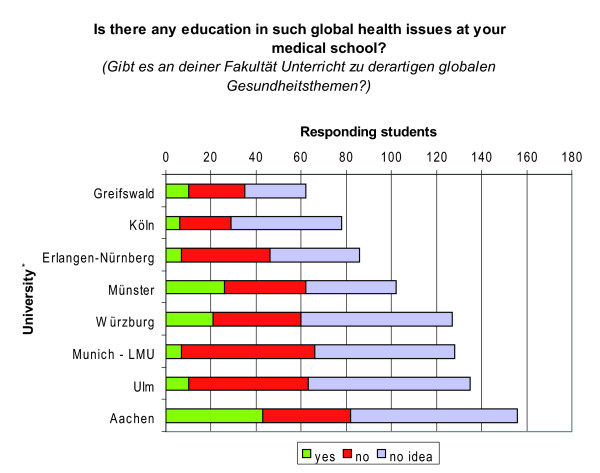
**Prevalence of global health education by medical school**. * Only universities with more than 50 respondents are illustrated.

Notably, however, responses of students enrolled at the same university were highly inconsistent (Figure 8) and no medical school was found, at which students unanimously affirmed the existence of education in global health. More consistent answers were only found at medical schools at which respondents either affirmed the absence of education in global health or had 'no idea' (e.g. Halle, Hamburg, Lübeck, Magdeburg, Mainz, Rostock, Saarbrücken). These universities were not illustrated in Figure 8 since less than 10 students respectively participated in our study (Additional File 1: Annex 1-University affiliation).

In view of these findings, conclusions on the prevalence of education in global health at institutional levels are highly questionable.

**Figure 9:**
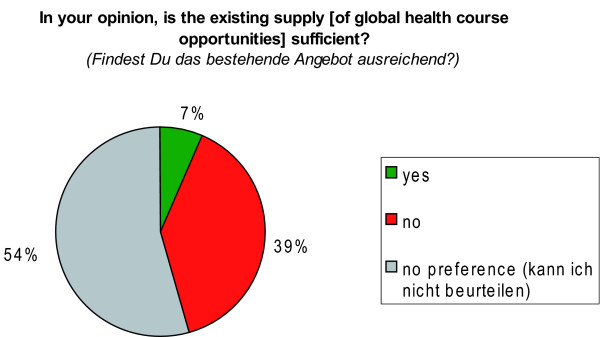
**Satisfaction with the supply of global health course opportunities**. N = 1126 students (100%).

Regarding the demand for learning opportunities in global health, 39.0% of the respondents felt that the existing supply is not sufficient. The majority of 54.0% felt they were not in a position to make judgements about the sufficiency of the supply and stated therefore 'no preference' (*'Kann ich nicht beurteilen'*) (Figure 9).

**Figure 10:**
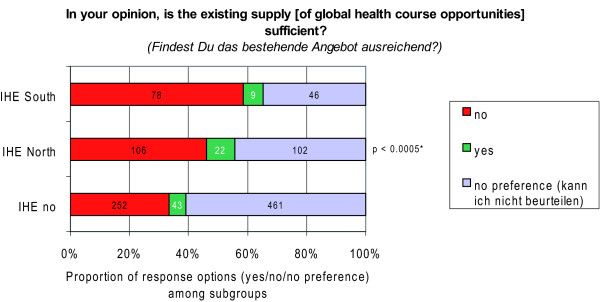
**Satisfaction with the supply of global health course opportunities by subgroups**. *p-value of Fischer's exact test. IHE = international health elective; IHE-South = electives predominantely completed in developing countries; IHE-North = electives predominantely completed in industrialised countries; IHE-no = no electives abroad.

Interestingly, the (dis-) satisfaction with the supply varied with a high statistical significance (p < 0.0005) between the different subgroups among our respondents. The proportion of students who were dissatisfied with the existing supply of global health course opportunities at their university was 13.0% higher among those, who completed their IHE predominantly in developing countries (IHE-South) compared to students who completed their IHE predominantly in industrialised countries (IHE-North). Similarly, the proportion of dissatisfied students was 25.0% higher among the subgroup IHE-South compared to students who had gathered no experiences abroad yet (IHE-no) (Figure 10).

**Figure 11:**
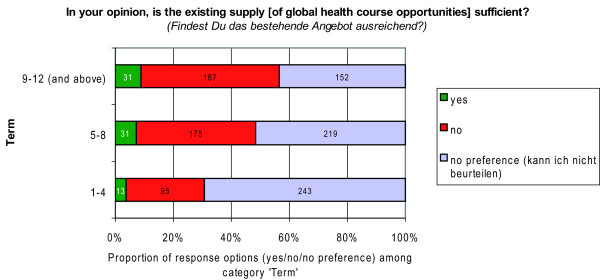
**Satisfaction with the supply of global health course opportunities by level of study**.

Regarding the different levels of study (Figure 11), the proportion of students who felt that the existing supply was insufficient rose from 27.0% among preclinical students (terms 1-4) to 48.0% among 5^th ^and 6^th ^year students (terms 9-12 and above). That means that nearly half of graduating students were dissatisfied with the supply of learning opportunities in global health. Whereas the proportion of those who felt the supply was sufficient rose about only 5.0% between students from preclinical (terms 1-4) and final clinical terms (terms 9-12 and above). (Figure 11)

**Figure 12:**
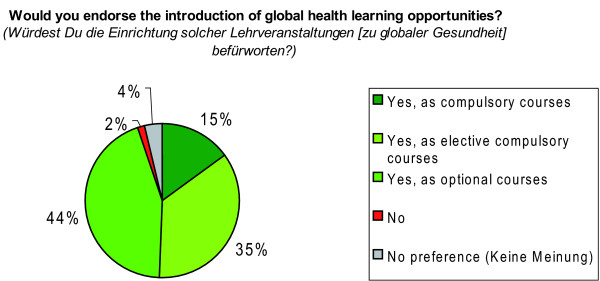
**Demand for global health learning opportunities**. N = 916 students (100%); n = 210 provided no answer.

**Figure 13:**
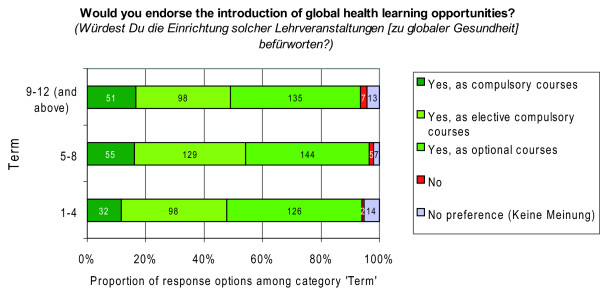
**Demand for global health learning opportunities by level of study**. N = 916 students (100%); n = 210 provided no answer.

Moreover, 94.0% of the respondents (N = 916) endorsed the idea of introducing global health learning opportunities as either compulsory, elective compulsory or optional courses, indicating a high demand for opportunities to learn about global health issues (Figure 12). The preferences varied only slightly, i.e. between 1.0% and 6.0%, for respective response options among students from different levels of study (Figure 13). For 210 participants responses were missing, most probably for technical reasons.

## Discussion

The purpose of this study was to assess the international health elective (IHE) patterns of medical students enrolled at German universities. We aimed to analyse whether or how this group prepares for their IHE and whether they are generally exposed to training courses in tropical medicine or global health. Finally, we assessed the prevalence of education in global health education at respondents' medical schools and analysed medical students' perceived needs and demands for education in global health.

Our findings provide evidence for a high mobility rate of 65.0% among graduating students in our sample (Figure 1) and prove that electives abroad are very common, notably also towards developing countries (Figure 2). One third of all responding students had already gained experiences abroad, many of them up to three times (Table 2), while 66.0% of those respondents without experiences abroad had plans for an IHE in the near future. These figures demonstrate a high mobility along with a high potential for future mobility among our sample.

More than one third of respondents with experiences abroad have completed their IHE predominantly in developing countries (Figure 2). While Rowson et al. hypothesised that IHEs, or other exchange opportunities appear to be the main gateway for acquiring some interaction with the field of global health for medical students in the UK *(Rowson M, Hughes R, Smith A, Maini A, Martin S, Miranda JJ, Pollit V, Wake R, Willott C, Yudkin JS: Global Health and medical education - definitions, rationale and practice, unpublished)*, we notice that this interrelation also works for medical students in our sample.

To our great surprise, preparation or training beyond self-study is virtually non-existent among our sample prior to international health electives (Table 3). The low participation rate in formal courses at universities may derive from a reluctance of students to utilise existing preparation courses. However, we believe it is more likely that targeted pre-elective training is not widely available for the majority of medical students in Germany. This argument is backed by consistent anecdotal evidence from the networks of the German Medical Students' Association and additionally by a recently conducted systematic review, which strongly suggests that international or global aspects are underrepresented in German medical curricula *(Bozorgmehr K, Tinnemann P: The State of Global Health in German Medical Education: a systematic review, unpublished)*. The picture produced by our sample parallels the situation in the UK described by Miranda et al., who revealed an absence of preparation opportunities for students prior to IHE apart from individual advice and travel health risk assessments [9].

The lack of immediate preparation seems to be compensated by students through their participation in other relevant learning opportunities. We found that mobile students were more frequently exposed to training courses in 'tropical medicine' or 'global health', especially when the destination was a developing country. As for the participation rate of 9.0% in global health courses (Table 2), we were not able to identify any comparable studies to estimate whether this prevalence is low or high compared to other countries. In view of the possibility that selection bias (inherent in the recruitment method of using mailing lists of student unions) might have influenced the participation rate of global health courses, we assume that the real participation rate in global health courses is considerably lower among the whole population of German medical students. The fact that the same electronic mailing-lists are used for distributing the announcements of our own global health courses adds to the likelihood that the real participation rate is lower. We can only speculate that courses done by the majority of respondents are those provided by student-led organisations (*Globalisation and Health Initiative*) or by other non-governmental organisations involved in non-formal education. Some learning opportunities in global health seem also to be existent at respondents' medical schools in different forms as stated by a small number of students, mainly as part of other compulsory courses (Figure 7).

Nevertheless, we refrain from drawing conclusions on the prevalence of education in global health at medical school levels based on these findings - mainly due to the highly inconsistent statements of students enrolled at the same university (Figure 8).

A recent systematic review of literature and curricula has shown that training in global health is not part of regular medical curricula in Germany *(Bozorgmehr K, Tinnemann P: The State of Global Health in German Medical Education: a systematic review, unpublished)*. The review explicitly considered the possibility that teaching in global health might occur as part of socio-medical or tropical medicine courses. Global health courses, however, which include the broader determinants of health were found to be part of non-formal education, either organised by student-led organisations [21], by other non-governmental organisations or as optional extra-curricular summer schools.

The dominant opinion in our sample was that the supply of learning opportunities at medical schools is not sufficient, especially amongst students with international experiences (Figure 10) and graduating students (Figure 11). This finding lends further credence to the above argument.

Notably, the dissatisfaction was higher among mobile students, especially when the destination was a developing country (Figure 10). This co-incidence suggests that students' mobility, especially towards developing countries, raises awareness of unmet educational needs and thus raise demands for educational interventions to meet those needs. But even among students without any experiences abroad, a respectable proportion (33.0%) was dissatisfied with the supply of learning opportunities in global health at their medical school. (Figure 10)

At the same time, we note a tremendous demand among our sample to create learning opportunities in global health as reflected by the fact, that 94.0% of 916 students endorsed the idea of introducing global health learning opportunities as either compulsory, elective compulsory or optional courses into their curricula (Figure 12). Interestingly, the illustrated demand for learning opportunities in global health among our sample is inconsistent with the low value placed by students on socio-medical subjects (e.g. social medicine) that has been shown in other studies among German medical students [22,23].

This contrast raises the question why education in global health receives such a high resonance among medical students, although the field deals with, e.g. social determinants of health, which could be also object of social medicine or public health (according to our definition: '*courses in which students analyse the influence on people's health of factors such as poverty, debt, globalisation, health systems and health financing, human rights, hunger, armed conflicts and migration')*. Taking into account that socio-medical subjects in German medical education such as social medicine too often lack a deep or critical engagement with the socio-political dimension of health, despite the fact that social and political issues created the field [24], one could speculate that classical teaching in socio-medical subjects does not assuage the 'appetite' of contemporary medical students to learn about the socio-political dimensions of health. Empirical evidence for this 'appetite' is provided by a study of Kuhlmey et al., who found that almost half of 1^st ^year medical students in Berlin showed an interest in socio-political issues and regarded these as 'extraordinary relevant' due to their future professional work as physicians [15]. If other educational interventions do not satisfy students' interest in this field, can education in global health induce or re-inforce students' appreciation or interest in socio-political issues? We will further elaborate on this question and provide some evidence for the potential benefits involved in education in global health in the second part of this article series [20].

Adding to the above, we were highly surprised by the low participation rate in courses of tropical medicine. We expected tropical medicine to be far more established in medical education than global health. In terms of the participation rate found in this study, we feel alarmed about the fact that a vast majority of 84.0% of all students (Table 2) and 72.6% of the 5^th ^and 6^th ^year students declared, that they have never participated in a course of tropical medicine (Table 4). Only 27.4% of 5^th ^and 6^th ^year students had already completed a course in tropical medicine, which means that about three quarters of all graduating students have never learnt in-depth about tropical diseases. Given a similar likelihood of selection bias as described above, a down-estimation of the participation rate - in our view - even aggravates the detrimental situation.

Based on the findings of a study in 1995, Stich et al. previously argued that tropical medicine and international health are systematically taught only in very few German universities *(Stich A, Köbler C, Strauß R, Hampel D, Fleischer K: Tropenmedizinische Ausbildung in Deutschland - Erfolge und Defizite: Teil 1-Lehrveranstaltungen zum Themenbereich "Tropenmedizin und Gesundheitsversorgung in Entwicklungsländern" an deutschen medizinischen Fakultäten, unpublished) *and hypothesised that the majority of graduates would never learn about Malaria beyond their courses in Medical Microbiology or Infectious Diseases *(Stich A, Diesfeld HJ, Fleischer K: Zum Stellenwert der Tropenmedizin an deutschen Universitäten, unpublished)*. The situation they have described seems to be similar to our findings (Table 4) twelve years later in 2007.

Notably, Stich and colleagues strongly doubted whether courses of Medical Microbiology or Infectious Diseases could impart the socio-political dimension of tropical diseases. Among all Institutes of Medical Microbiology and Hygiene (N = 37) questioned in 1995/1996 by Stich et al., the topics "control of endemic tropical diseases", "health care management and public health" as well as "root causes of illness and poverty" were reported to be 'only outlined' or 'not covered' by the majority of institutes. *(Stich A, Köbler C, Strauß R, Hampel D, Fleischer K: Tropenmedizinische Ausbildung in Deutschland - Erfolge und Defizite: Teil 1-Lehrveranstaltungen zum Themenbereich "Tropenmedizin und Gesundheitsversorgung in Entwicklungsländern" an deutschen medizinischen Fakultäten, unpublished)*. A similar analysis of the global health topics found to be provided "as part of compulsory courses" in our study (Figure 7) would be interesting, to assess whether these courses impart respective topics as comprehensive as full courses.

Besides the low exposure to courses in global health and tropical medicine, we have analysed perceived needs and have identified a gap between students' mobility and their formal preparation prior to IHE. Educational shortcomings due to the low exposure to the examined educational interventions and due to the preparation gap are not determined yet. Whether or not the indentified gap and the unmet perceived needs lead to inadequate care in practice was beyond the scope of this survey, but is certainly an interesting and important issue for further research.

Without attempting to generalise our findings, however, we note that they lend further credence to critical voices arguing that formal medical education in Germany does not prepare its future health workforce adequately for living and working in the global village *(Bozorgmehr K, Tinnemann P: The State of Global Health in German Medical Education: a systematic review, unpublished)*.

Beyond educational shortcomings, there are ethical questions to be taken into account as well. The fact that German medical students work in foreign countries without being prepared for the local health conditions and diseases may represent a danger for those patients who are depending on them; especially in low-resource settings with a shortage of human resources for health and lack of supervision. In some cases overestimation of the capabilities of the 'foreign doctor' might add to the conflict.

This potential conflict is illustrated by the comment of a student, who provided the following statement in the free text options regarding the purpose of his/her IHE in South Africa: 'In surgery. Was totally amazing! I can recommend it to everyone who is interested in surgery, because there you can easily do anything what you dare to do!' *('In der Chirurgie. War total super! Kann ich nur jedem empfehlen, der an der Chirurgie interessiert ist, weil man da einfach alles machen darf, was man sich selber zutraut')*. This 25 years old student was one of those 131 students with experiences abroad, who did not prepare specifically at all (Table 3).

This statement leaves evolving questions about ethical aspects of students' attitudes and practices abroad unanswered. In particular, it questions the existence of mechanisms, which mitigate adverse effects by the lack of preparation (e.g. of social or cultural nature) on the host community as well as on students themselves.

On the other hand, we believe that an adequate preparation of students regarding social, cultural, economic and political influences on health before their IHE might in return increase the educational effect of an IHE and sensitise students to the non-biomedical causes of ill health abroad [20] and even within their own country.

In this context, we speculate that nothing exists to process students' experiences and impressions abroad upon returning to their regular studies. This gap obviously suggests potential possibilities to channel the educational benefits made during an IHE within the German medical (education) system.

Embedding preparation modules in a comprehensive programme of teaching global health, e.g. as proposed by the German Medical Students' Association [25] could help to fill gaps both in preparation and debriefing. Targeted pre-elective preparation and comprehensive post-elective courses in global health, which build on the experiences gained abroad, could avoid that IHE are solely (ab-) used as 'international holiday electives', become a trendy 'must-have experience' or a part of medical tourism from North to South.

We believe that if our findings regarding students' mobility and their perceived needs and demands for the introduction of global health learning opportunities would apply to all students in Germany, it would be necessary to advance future curricula by adjusting them to a globalising world. Thereby, potential educational effects involved in IHE could be enhanced and used as an educational window for a further engagement with 'influences of factors such as poverty, debts, globalisation, health systems and health financing, human rights, hunger, armed conflicts and migration on people's health'.

But it is very important to note that medical students' interest or disinterest in subjects, e.g. in the case of students' disinterest in epidemiology or social medicine in Germany [22,23], does not in itself either increase or reduce the importance of these subjects, nor does their interest or disinterest alone justify the inclusion or exclusion of subjects from medical education. Therefore, we acknowledge that the sole demand for learning opportunities in global health expressed by our sample is not enough to argue for their introduction into curricula. Therefore, we will analyse knowledge gaps among our sample related to global health issues and present potential benefits involved in students' mobility and education in global health or tropical medicine in the second part of this article [20].

## Limitations and strengths

The study design we applied to answer the research questions bears several weaknesses. Recruiting participants by using mailing-lists of students' unions bears the possibility that individuals responded more than once to our survey, e.g. if the student was on multiple lists. The survey software we used had no option to avoid this without conflicting with the anonymity of the survey.

The recruitment method may also imply selection bias towards 'especially motivated' students regarding their participation rate in examined courses or regarding their mobility. Due to our recruitment method, which attempted to reach as many students as possible, we received an opportunistic sample with a skew in originating schools. Thus, we can determine response rates (1.4%) only related to the whole population of medical students in Germany (78.067). Consequently, the findings presented in this paper are not generalisable. Due to the fact that the collected data was skewed to several schools, drawing conclusions about individual schools is similarly not directly possible. Therefore, any conclusions refer to the analysed sample only.

In terms of the high student mobility, however, our findings are consistent with findings of other studies conducted in Germany [14,15]. Moreover, if our sample would predominantly include 'especially motivated' students, the lack of preparations prior to IHE (Table 3) and the low participation rate in courses of tropical medicine amongst our sample would be even more alarming. In fact, we would wonder how 'unmotivated students' do prepare prior to their IHE if our findings reflect the preparation of the 'especially motivated' students.

Detailed factors related to the settings of an IHE were not captured by our questionnaire. Therefore, the stratification criteria applied to group medical students by the destination countries of their IHE were too crude to capture disparities between countries and settings. Students who completed an IHE in an industrialised country might nevertheless have been working in an underserved or low-resource area and students who declared an IHE in a developing country might have worked in a high-standard, high-resource setting, e.g. in private instead of governmental hospitals. Other stratification criteria, such as participation in courses of global health or tropical medicine, were also not specified in terms of course duration and course providers.

It could be considered premature to ask students of all terms about their perceived needs and demands regarding curricular contents, since younger students might not have a well-founded appreciation for what knowledge, skills, and attitudes they require in order to be physicians. However, from a participative perspective, asking students of all levels what their curriculum should include is essential to learn more about their interests and perceived needs.

We also asked medical students of all terms in order to determine the prevalence of global health education at their medical school. Unfortunately, replies from students enrolled at the same university were highly inconsistent, which makes sound conclusions on the prevalence very difficult. However, asking only graduating students or physicians in practice might similarly have biased conclusions on the prevalence of global health education, since reforms for younger students might in the meantime have changed curricula, thereby filling the educational gaps identified by the older and ex-students.

The inconsistent replies regarding the prevalence of global health courses might also be a result of the absence of a definition of 'education' in our questionnaire when asking students about 'education in global health' at their medical school. The imprecise use of this term might have led students to answer the question related to the existence of global health courses at their institution with 'yes', even if the defined topics (*'influences of factors such as poverty, debts, globalisation, health systems and health financing, human rights, hunger, armed conflicts and migration on people's health'*) were solely part of other subjects, e.g. being touched on by courses in social medicine, epidemiology, or medical sociology, etc. As Figure 7 shows, this was the case in 31.0% of replies regarding the form of education in global health. The authors however agree with Stich et al., that the socio-political dimension of global health or topics related to tropical medicine cannot be adequately covered solely as part of other courses, which was the rationale behind asking for full courses when we analysed the exposure to the courses of interest.

Legitimate conclusions in the context of course existence are that either there is a lack of information about curricular content at respective institutions or that students did not seek existing information before answering the questionnaire, since nearly half of questioned students (47.0%) simply had 'no idea', when asked about the existence of education in global health at their medical school.

It remains unclear, whether the lack of information is caused by a poor communication of curricular contents between faculty and students, by lacking engagement of students with their own curricula or rather by the pure absence of learning opportunities in this field. A similar phenomenon was observed in Finland by Matin in 2003 *(unpublished)*, who noted that 'the majority of students in four of the five universities did not know whether there currently was global health teaching at their faculty'.

Beyond doubt, a better approach to determine the prevalence of global health education would have been to question deans of medical schools. However, international surveys conducted in 2005 *(Sundell T, Ashorn P: International Health in Medical Curricula: Report of an International Survey, unpublished) *and 2007 *(Rowson M, Hughes R, Smith A, Maini A, Martin S, Miranda JJ, Pollit V, Wake R, Willott C, Yudkin JS: Global Health and medical education - definitions, rationale and practice, unpublished) *addressed deans of German medical schools and have produced unsatisfying response rates due to the lack of cooperation by medical schools. Therefore, a repetition of a similar approach in such a short period seemed inappropriate to us back in 2007.

Asking students of all terms whether they 'would endorse the introduction of global health learning opportunities' revealed valuable insights into students' opinions towards global health education (Figure 13). However, the wording of the question and the provided answer options (Figure 12) might have produced courtesy replies. Asking for the importance students place on the introduction of global health courses and providing answer options on an e.g. 1-to-6 scale might have produced more differentiated replies.

Although the overall response rate amongst the total number of German medical students might be considered low (1.4%), we believe that we received a sufficiently large number of responses (N = 1126) allowing us to draw conclusions among our sample about mobility patterns, their satisfaction with the current supply of global health teaching and demand for creating more learning opportunities. We further believe that the conclusions drawn from this sample might be more comprehensive than from a hypothetical sample with higher relative size, but considerably smaller absolute size.

Notably, the quantity of research analysing educational needs of German medical students in general [26], and especially in the context of a globalising world, is poor and virtually non-existent in Germany *(Bozorgmehr K, Tinnemann P: The State of Global Health in German Medical Education: a systematic review, unpublished)*. Therefore we believe that it is especially important to identify educational needs under a changing environment - e.g. raised by the increasing mobility of people - and to identify ways and educational windows to address these needs. This study adds evidence to existing gaps in these areas and portrays student perspectives in discussions about global health in medical education. Backed by other works *(Bozorgmehr K, Tinnemann P: The State of Global Health in German Medical Education: a systematic review, unpublished)*, we can state that our study could be regarded as the first structured attempt since 1995 to analyse the implications for German medical education raised by aspects of globalisation.

As an important area for future research, we would like to stress that more detailed and specific needs assessments should be conducted among students and practitioners to identify educational needs in the context of globalisation.

## Conclusion

We have provided evidence of a high mobility rate among graduating medical students in our sample and have shown that international health electives (IHE) in developing countries make up nearly one third of all international rotations. Formal preparation prior to IHE beyond self-study was exceptional among our analysed sample. The general exposure to relevant educational interventions in tropical medicine or global health was appallingly low (16.0% vs. 9.0%), also among 5^th ^and 6^th ^year students (27.4% vs. 12.2%). Our findings support previous arguments that German medical education lacks opportunities to learn about tropical medicine and international or global health issues; despite expressed needs and demands among medical students in our sample. We were not able to definitely determine the prevalence of global health education at German medical schools, but we revealed a high dissatisfaction with the supply of learning opportunities in global health among our sample, especially among mobile and graduating medical students. This perceived need for more learning opportunities in global health was accompanied by an unequivocal demand for the introduction of global health into medical curricula.

Despite the limitations of our findings we recommend that medical schools and public health educators consider students' mobility and their exposure to various health care systems in future curricular reforms, and actively engage in preparation before and education after international health electives. Responding to students' demands, instructional reforms should embed such attempts in comprehensive and structured education in global health.

## Competing interests

### Financial competing interests

KB, KS and JMS are members of the Globalisation and Health Initiative (GandHI) of the German Medical Students' Association (Bundesvertretung der Medizinstudierenden e.V.). GandHI receives fundings by Capacity Building International (InWEnt)/Federal Ministry for Economic Cooperation and Development (BMZ). This study was financially supported by the German Medical Students' Association, the article-processing charge was covered by the Institute for Social Medicine, Epidemiology and Health Economics, Charité - University Medical Centre Berlin.

The authors declare that they have no financial competing interests.

### Non-financial competing interests

This study has been conducted as part of the research thesis of KB to gain an academic degree *(Dr.med) *at the Institute for Social Medicine, Epidemiology and Health Economics, Charité - University Medical Center Berlin, Germany. Although grateful for the financial support by the German Medical Students' Association, the authors attest that the conclusions in this study are theirs alone and not necessarily endorsed by the Globalisation and Health Initiative (GandHI) or the German Medical Students' Association (Bundesvertretung der Medizinstudierenden e.V.).

## Authors' contributions

All authors have made substantial contributions to the study conception and design. KS and JMS contributed significantly to designing the questionnaire, acquisition of data and revising drafts of the manuscript. KB developed the hypotheses, performed the statistical analysis and evaluated the data, produced figures and tables and drafted and revised the manuscript in light of the peer-reviewers' comments. PT was involved in all steps of the study and provided critical advice during the conduct and evaluation of this study. All authors have read and approved the final manuscript.

## Pre-publication history

The pre-publication history for this paper can be accessed here:

http://www.biomedcentral.com/1472-6920/10/66/prepub

## Supplementary Material

Additional file 1 **Annex 1-University affiliation**. N = 1126 students (100%). illustrates the university affiliation of all responding students. Click here for file

## References

[B1] Scholte JA What Is Globalization? The Definitional Issue - Again 2002 Coventry, Centre for the Study of Globalisation and Regionalisation (CSGR), Department of Politics and International Studies, University of Warwick CSGR Working Paper 109/02

[B2] Huynen MMTE Martens P Hilderink H The health impacts of globalisation: a conceptual framework Globalization and Health 2005 1 14 10.1186/1744-8603-1-14 16078989 PMC1208931

[B3] Bateman C Baker T Hoornenborg E Ericsson U Bringing global issues to medical teaching Lancet 2001 358 1539 1542 10.1016/S0140-6736(01)06586-2 11705584

[B4] Editorial Educating doctors for world health Lancet 2001 1471 11705551

[B5] Edwards R Rowson M Piachaud J Teaching international health issues to medical students Med Educ 2001 35 807 808 10.1046/j.1365-2923.2001.1014c.x 11489114

[B6] Haq C Rothenberg D Gjerde CL New World Views: Preparing Physicians in Training for Global Health Work Fam Med 2000 32 566 572 11002868

[B7] Drain PK Primack A Hunt DD Fawzi WW Holmes KK Gardner P Global health in medical education: a call for more training and opportunities Acad Med 2007 82 226 230 10.1097/ACM.0b013e3180305cf9 17327707

[B8] Houpt ER Pearson RD Hall TL Three domains of competency in global health education: recommendations for all medical students Acad Med 2007 82 222 225 10.1097/ACM.0b013e3180305c10 17327706

[B9] Miranda JJ Yudkin JS Willott C International Health Electives: Four years of experience Travel Med Infect Dis 2005 3 133 141 10.1016/j.tmaid.2004.09.003 17292031

[B10] Edwards R Piachaud J Rowson M Miranda J Understanding global health issues: are international medical electives the answer? Med Educ 2004 38 688 690 10.1111/j.1365-2929.2004.01849.x 15200392

[B11] Izadnegahdar R Correia S Ohata B Kittler A ter KS Vaillancourt S Global health in Canadian medical education: current practices and opportunities Acad Med 2008 83 192 198 10.1097/ACM.0b013e31816095cd 18303368

[B12] Reali C Mapping of undergraduate courses on Global Health in Italy, assessment of needs, and analysis of training opportunities Equal opportunities for Health: Action for Development 2007

[B13] Johnson O Baker C Medsin Survey of Interest in Global Health amongst King's Medical Students 2008 London, Medsin King's

[B14] Federkeil G CHE Alumni-Ranking Medizin Ergebnisse einer vergleichenden Absolventenbefragung Humanmedizin des Centrums für Hochschulentwicklung 2004 http://www.che.de

[B15] Kuhlmey A Dettmer S Karriere- und Lebensplanung in der Medizin: Studierende, Absolventen und junge Ärzte im Wandel des Gesundheitssystems 2007 http://www.charite.de18027518

[B16] Heublein U Özkilic M Sommer D Aspekte der Internationalität deutscher Hochschulen. Internationale Erfahrungen deutscher Studierender an ihren heimischen Hochschulen Band 63 2007 Bonn, DAAD, Deutscher Akademischer Austauschdienst. Dokumentationen & Materialien

[B17] ÄAppO Approbationsordnung für Ärzte vom 27.Juni 2002 (BGB1. I S.2405), die zuletzt durch Artikel 7 des Gesetzes vom 30. Juli 2009 (BGB1. I S. 2495) geändert worden ist. BGB1. I, 2405 2002

[B18] Statistisches Bundesamt Studierende an Hochschulen - Vorbericht Fachserie 11, Reihe 4.1, 21 2008 Wiesbaden. Bildung und Kultur

[B19] Chenot JF Undergraduate medical education in Germany GMS Ger Med Sci 2009 7 Doc02 10.3205/000061 PMC2716556 19675742

[B20] Bozorgmehr K Menzel-Severing J Schubert K Tinnemann P Global Health Education: a cross-sectional study among German medical students to identify needs, deficits and potential benefits (Part 2 of 2: Knowledge gaps and potential benefits) BMC Medical Education 2010 10 67 10.1186/1472-6920-10-67 20932278 PMC2958968

[B21] Bozorgmehr K Oezbay J GandHI - The German response to deficits in medical education The Lancet Student 2008

[B22] Jansen M Verbesserungspotenzial des Medizinstudiums und Relevanz der Fachgebiete aus retrospektiver Sicht der Fachärzte 2007 Düsseldorf: Medizinische Fakultät der Heinrich-Heine-Universität (HHU), Anatomisches Institut II

[B23] Jungbauer J Kamenik C Alfermann D Brahler E How do young physicians assess their medical studies in retrospect? Results of a medical graduates' survey in Germany Gesundheitswesen 2004 66 51 56 10.1055/s-2004-812705 14767791

[B24] Schagen U Scope of social medicine 20 years after adoption into medical education--analysis test questions 1976-1996 by the IMPP (Institute for Medical and Pharmaceutical Test Questions) Gesundheitswesen 1998 60 13 20 9522558

[B25] Bozorgmehr K Last K Müller A Schubert K Cutting-Edge Education - Global Health in Medical Training: Proposals, Educational Objectives and Methodological Recommendations GMS Z Med Ausbild 2009 26

[B26] Hahn EG Medical Education Research in German Speaking Countries: Quantité Négligable? GMS Z Med Ausbild 2005 22

