# Supplementary figures and images for: Global Health Education: a cross-sectional study among German medical students to identify needs, deficits and potential benefits (Part 1 of 2: Mobility patterns & educational needs and demands)
Source: BMC Med Educ. 2010 Oct 8;10:66. doi: 10.1186/1472-6920-10-66 (PMC2958967; doi:10.1186/1472-6920-10-66)

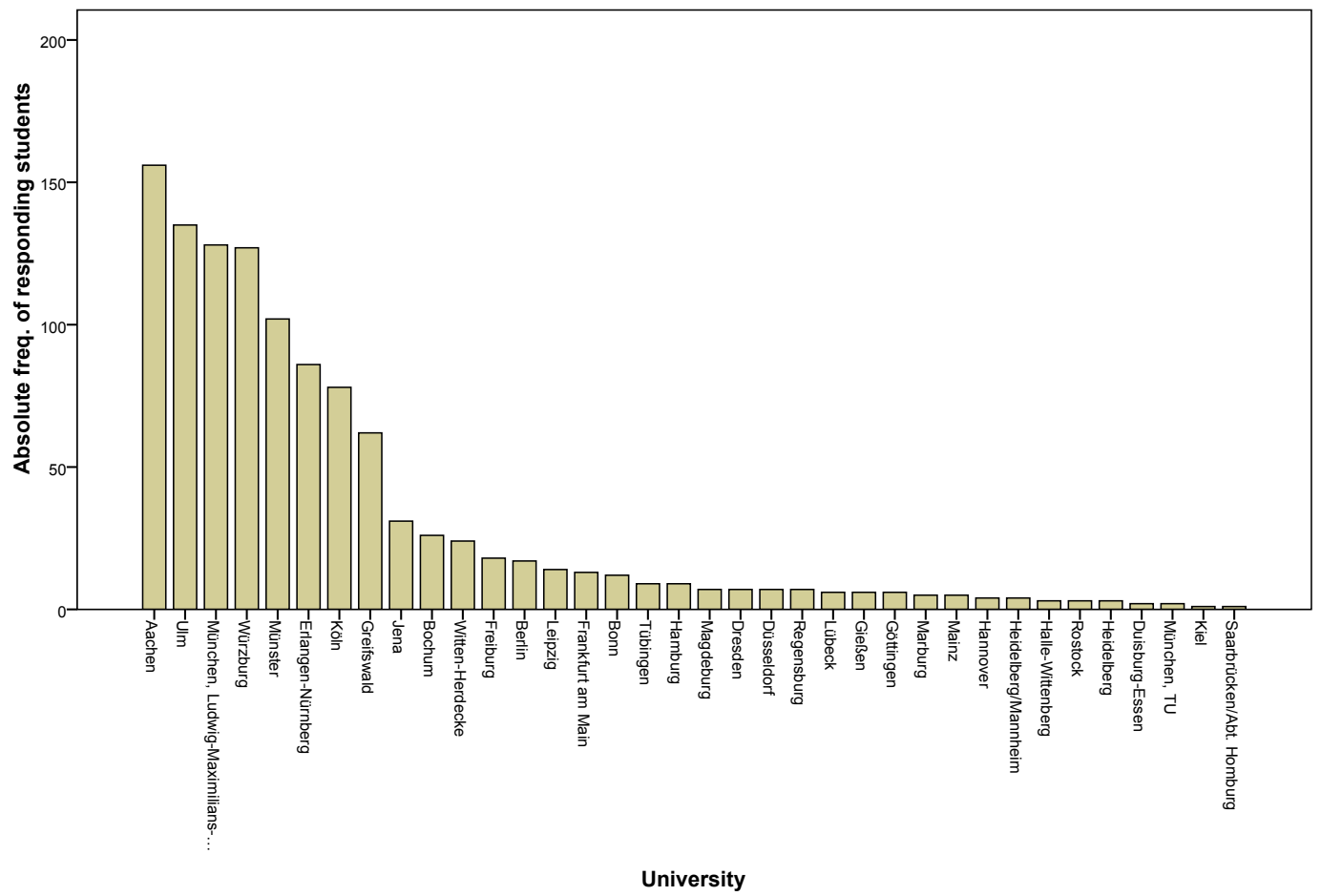

Supplement: Additional file 1 — Annex 1-University affiliation. N = 1126 students (100%). illustrates the university affiliation of all responding students. [file 1472-6920-10-66-S1.PDF]
